# Engineering and systems-level analysis of *Pseudomonas chlororaphis* for production of phenazine-1-carboxamide using glycerol as the cost-effective carbon source

**DOI:** 10.1186/s13068-018-1123-y

**Published:** 2018-05-04

**Authors:** Ruilian Yao, Keli Pan, Huasong Peng, Lei Feng, Hongbo Hu, Xuehong Zhang

**Affiliations:** 1 State Key Laboratory of Microbial Metabolism, and School of Life Sciences and Biotechnology, Shanghai Jiao Tong University, 800 Dongchuan Road, Shanghai, 200240 China; 2 Instrumental Analysis Center, Shanghai Jiao Tong University, 800 Dongchuan Road, Shanghai, 200240 China

**Keywords:** *Pseudomonas chlororaphis*, Phenazine-1-carboxamide, Glycerol, Metabolomics, ^13^C, Channeling

## Abstract

**Background:**

Glycerol, an inevitable byproduct of biodiesel, has become an attractive feedstock for the production of value-added chemicals due to its availability and low price. *Pseudomonas chlororaphis* HT66 can use glycerol to synthesize phenazine-1-carboxamide (PCN), a phenazine derivative, which is strongly antagonistic to fungal phytopathogens. A systematic understanding of underlying mechanisms for the PCN overproduction will be important for the further improvement and industrialization.

**Results:**

We constructed a PCN-overproducing strain (HT66LSP) through knocking out three negative regulatory genes, *lon*, *parS*, and *prsA* in HT66. The strain HT66LSP produced 4.10 g/L of PCN with a yield of 0.23 (g/g) from glycerol, which was of the highest titer and the yield obtained among PCN-producing strains. We studied gene expression, metabolomics, and dynamic ^13^C tracer in HT66 and HT66LSP. In response to the phenotype changes, the transcript levels of *phz* biosynthetic genes, which are responsible for PCN biosynthesis, were all upregulated in HT66LSP. Central carbon was rerouted to the shikimate pathway, which was shown by the modulation of specific genes involved in the lower glycolysis, the TCA cycle, and the shikimate pathway, as well as changes in abundances of intracellular metabolites and flux distribution to increase the precursor availability for PCN biosynthesis. Moreover, dynamic ^13^C-labeling experiments revealed that the presence of metabolite channeling of 3-phosphoglyceric acid to phosphoenolpyruvate and shikimate to trans-2,3-dihydro-3-hydroxyanthranilic acid in HT66LSP could enable high-yielding synthesis of PCN.

**Conclusions:**

The integrated analysis of gene expression, metabolomics, and dynamic ^13^C tracer enabled us to gain a more in-depth insight into complex mechanisms for the PCN overproduction. This study provides important basis for further engineering *P*. *chlororaphis* for high PCN production and efficient glycerol conversion.

**Electronic supplementary material:**

The online version of this article (10.1186/s13068-018-1123-y) contains supplementary material, which is available to authorized users.

## Background

**Fig. 1:**
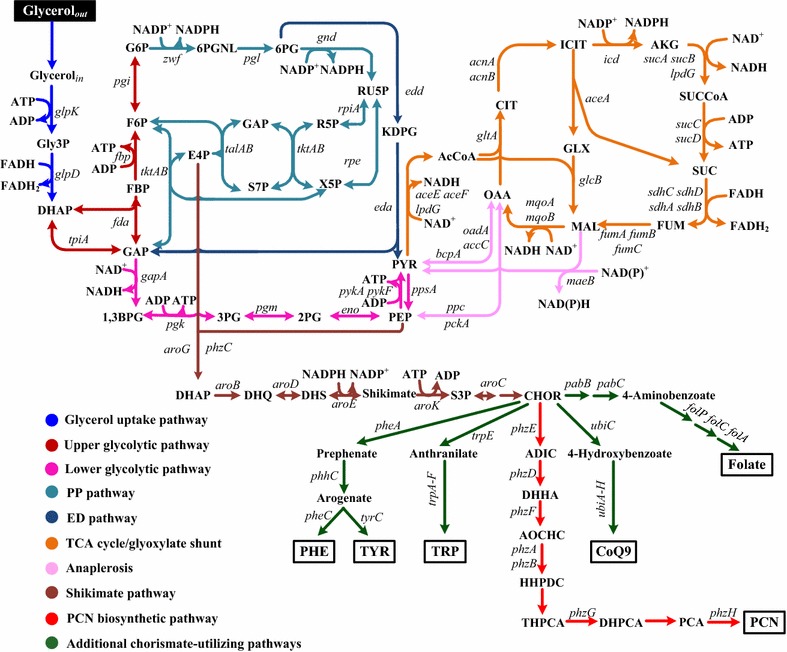
Metabolic pathways for PCN biosynthesis in *P. chlororaphis*. Broken lines illustrate multiple steps

In recent years, the fast development of biofuel and bioethanol industries has led to the production of large quantities of waste glycerol [1]. Significant amounts of glycerol surplus have given rise to a sharp drop in the glycerol price, making it a promising industrial carbon source for the production of value-added chemicals [2, 3]. Glycerol can be used to synthesize phenazine-1-carboxamide (PCN), a phenazine derivative, by *Pseudomonas* strains [4–6]. Phenazines are heterocyclic, nitrogen-containing compounds, which have attracted attention due to their broad spectrum antibiotic properties and roles in virulence, and been widely used in the biological control of various fungal phytopathogens [7]. PCN is one of the important molecules among phenazine compounds [8]. Figure 1 shows the metabolic pathways for PCN biosynthesis in *Pseudomonas chlororaphis* grown on glycerol. The gene cluster *phzABCDEFGH* is responsible for PCN biosynthesis [8, 9]. The production of PCN is regulated by different groups of genes: the *phzI*/*phzR* quorum-sensing system [10], the *gacS*/*gacA* regulatory genes [11], the *parS*/*parR* regulatory genes [12], and the *psrA* gene [13]. PhzI produces the autoinducer *N*-hexanoyl-l-homoserine lactone (C6-HSL), which binds to PhzR. Subsequently, the PhzR-C6-HSL complex probably binds to the upstream of *phz* operon, resulting in the initiation of this operon [10, 14]. The GacS/GacA two-component system is a master regulator of secondary metabolism, stimulates the production of phenazines in *Pseudomonas chlororaphis* and other *Pseudomonas* species [15, 16]. In contrast to PhzI/PhzR and GacS/GacA, ParS/ParR and PsrA act as repressors for the production of PCN [13]. Modifying these regulatory genes led to the improvements of the PCN titer and yield [10, 11, 13]. However, until now, underlying mechanisms for the PCN overproduction still remain obscure, which have been the bottleneck for the further improvement and industrialization. To fully understand the mechanisms, it requires further quantifying and integrating the metabolic function, regulation, and the physiological parameters at a systematic level.

Metabolomics, comprehensive analysis of a wide range of metabolites, is a broad and sensitive method to detect differences in metabolic states between conditions [17]. Combining metabolite levels with dynamic ^13^C-labeling is a powerful tool to peak inside the black box of the cellular metabolism, even in the absence of quantitative flux analysis [18]. It can provide useful information on the relative pathway activities and qualitative changes in pathway contributions, identify bottlenecks for the target product synthesis within the linear pathways, discover the latent pathway interactions, and help infer fluxes [19–21].

In our previous work, *Pseudomonas chlororaphis* HT66 isolated from the rice rhizosphere produced 0.42 g/L of PCN in King’s B medium [6]. In this study, we generated PCN-overproducing *P. chlororaphis* starting from HT66 by deleting regulatory genes. To interrogate the metabolism for the PCN overproduction and enable metabolic engineering approach, we studied gene expression, metabolomics, and dynamic ^13^C tracer in the base strain and the engineered strain. To the best of our knowledge, this is the first study to evaluate metabolic responses to the PCN overproduction on different organizational levels.

## Results

### Construction of PCN-overproducing strains

**Fig. 2:**
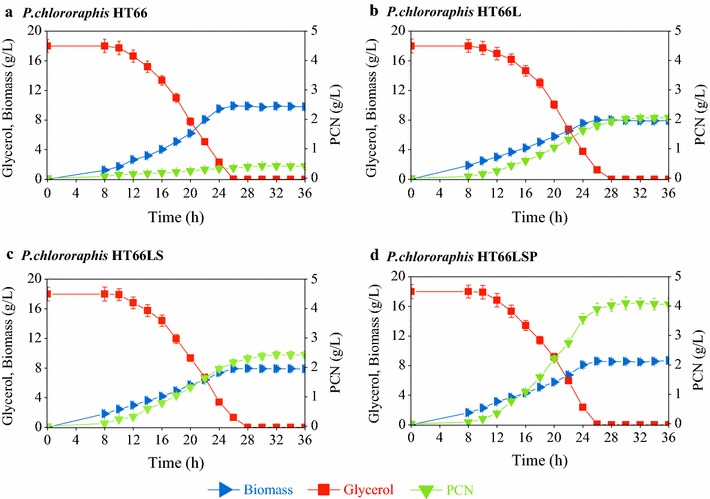
Culture profiles of PCN-producing *P. chlororaphis* strains HT66 (**a**), HT66L (**b**), HT66LS (**c**), and HT66LSP (**d**). Data represent the mean ± SD from three independent cultures

The GacS/GacA two-component system is a key element in the Gac/Rsm cascade [22]. This cascade activates the production of phenazine [23], which is negatively regulated by Lon protease [24]. It was reported that the Δ*lon* mutant showed elevated antibiotic production in *P. protegens* [24]. Thus, the strain HT66L was constructed by the disruption of *lon* in HT66. Disruption of *lon* diminished the maximum biomass (*X*_max_) produced by 19.0% and the specific growth rate (*μ*) by 27.2%, but did not cause significant changes to the specific glycerol consumption rate (Table 1). As expected, HT66L produced 2.05 g/L of PCN (Fig. 2b), which was 4.9 times of that produced in the base strain (Fig. 2a).

**Table 1:** Growth kinetic parameters of PCN-producing *P. chlororaphis* strains

Strain	Specific growth rate (h^−1^)	Specific glycerol consumption rate (mmol/g/h)	Specific PCN production rate (mmol/g/h)	PCN titer (g/L)	*Y*_P/G_ (mol/mol)
HT66	0.11 ± 0.003	2.51 ± 0.07	0.01 ± 0.0006	0.42 ± 0.02	0.01 ± 0.001
HT66L	0.08 ± 0.003	2.51 ± 0.07	0.10 ± 0.002	2.05 ± 0.10	0.05 ± 0.002
HT66LS	0.08 ± 0.003	2.52 ± 0.07	0.13 ± 0.003	2.43 ± 0.12	0.05 ± 0.002
HT66LR	0.08 ± 0.003	2.51 ± 0.06	0.11 ± 0.002	2.22 ± 0.11	0.05 ± 0.002
HT66LSP	0.08 ± 0.003	2.51 ± 0.07	0.23 ± 0.005	4.10 ± 0.21	0.09 ± 0.005

*Y*_*P/G*_ PCN yield on glycerol

The ParS/ParR two-component system, which consists of a membrane-bound histidine sensor kinase (ParS) and a cytoplasmic response regulator (ParR), plays a negative regulatory role in phenazine biosynthesis [12, 25]. Deletion of *parS* or *parR* led to increased pyocyanin production [12]. In order to gain more PCN, *parS* was deleted from HT66L to construct HT66LS, and *parR* was deleted from HT66L to construct HT66LR. The specific growth rates and the specific glycerol consumption rates in HT66LS and HT66LR were comparable with HT66L (Table 1). The PCN productions in HT66LS and HT66LR were increased to 2.43 and 2.22 g/L (Fig. 2c; Additional file 1), 5.9 and 5.3 times higher than that in HT66 (Fig. 2a). Thus, HT66LS was used for the further studies.

PsrA belongs to the TetR/AcrR family of transcriptional regulators, which inhibits the PCN production in *P. chlororaphis* PCL1391 in rich growth medium [13]. In order to further enhance the PCN production, the *prsA* gene was knocked out in HT66LS, and the resulting strain was named HT66LSP. HT66LSP exhibited a 13.1% decrease in the *X*_max_ and a 27.2% decrease in μ compared with HT66 (Table 1). The glycerol consumption rate kept constant. The strain HT66LSP produced 4.10 g/L of PCN at a yield of 0.23 (g/g) from glycerol after 30 h (Fig. 2d; Table 1), which represents, to our knowledge, the highest PCN titer and the yield ever obtained among engineered PCN-producing strains. Meanwhile, the specific PCN production rate was also improved in HT66LSP (Table 1). To determine the metabolic responses to the PCN overproduction, HT66 and HT66LSP were selected for a comparative analysis on different organizational levels.

### Relative gene transcription levels

**Fig. 3:**
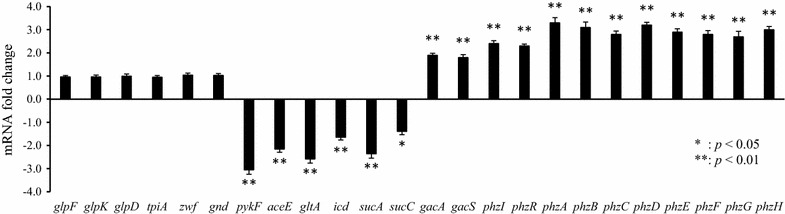
Fold changes of transcription levels of selected genes in *P. chlororaphis* HT66LSP compared with HT66

To investigate the transcriptional responses to the PCN overproduction, we analyzed the transcription levels of the HT66LSP genes by qRT-PCR, and compared the data with those of HT66 (Fig. 3). Compared with HT66, the expression levels of *glpF*, *glpK*, and *glpD* in the glycerol utilization pathway, *tpiA* in glycolysis, and *zwf* and *gnd* in the oxidative pentose phosphate (PP) pathway were not significantly altered in HT66LSP. In addition, the transcription levels of *pykF* and *aceE* in the lower glycolytic pathway, and *gltA*, *icd*, *sucA*, and *sucC* in the TCA cycle were downregulated in HT66LSP. Due to the deletion of *lon*, the transcript levels of *gacA* and *gacS* were upregulated in HT66LSP compared with the wild-type strain, confirming its key role for PCN synthesis. In addition, the transcription of autoinducer synthase and transcriptional activator genes *phzI* and *phzR* increased in HT66LSP. In accordance with the activation of *phzI*/*phzR*, the transcript levels of *phz* biosynthetic genes were all upregulated in HT66LSP, which could explain the observed high PCN production. The overexpression of *phzI*/*phzR* and the *phz* biosynthetic operon in a *psrA* mutant of *P. chlororaphis* PCL1391, and the upregulation of *phzA*, *phzB*, and *phzH* in a *parS* mutant of *P. aeruginosa* have been previously reported [12, 13]. These results were consistent with our findings.

### Metabolome analysis

**Fig. 4:**
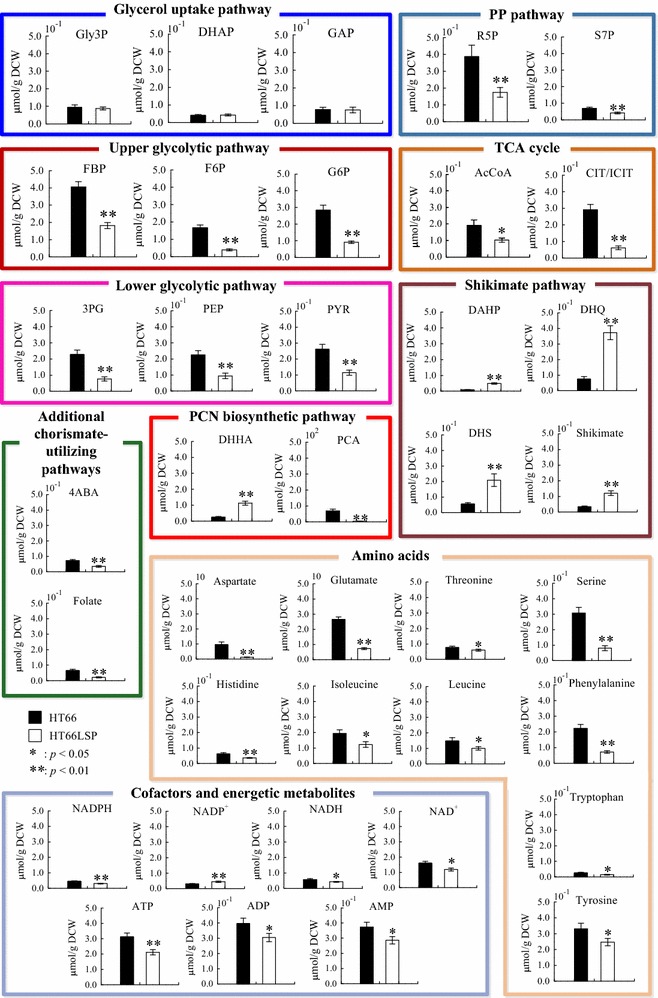
Comparison of intracellular metabolite concentrations of *P. chlororaphis* strains HT66 and HT66LSP. Data represent the mean ± SD from five independent cultures

To gain a further insight into mechanisms for the PCN overproduction, comparative metabolomics approach was employed to analyze metabolite concentrations difference of HT66 and HT66LSP (Fig. 4). A total of 38 metabolites were determined by isotope-assisted LC–MS, including central carbon metabolites, shikimate pathway metabolites, PCN biosynthetic pathway metabolites, chorismate-utilizing pathways metabolites, amino acids, and cofactors.

In the glycerol uptake pathway, the pool sizes of Gly3P, DHAP, and GAP were nearly constant for the two strains, indicating their pool sizes were strictly controlled. Central carbon metabolites in glycolysis (FBP, F6P, G6P, 3PG, PEP, and PYR), the PP pathway (R5P and S7P), and the TCA cycle (AcCoA and CIT + ICIT) were present in significantly lower concentrations in HT66LSP than in HT66. In addition, the intracellular concentrations of AKG, SUC, and FUM downstream of ICIT were below detection levels. It was reported that a low activity of the enzymes within the reductive TCA cycle and a high activity of the glyoxylate shunt were observed in *P. putida* KT2440 when grown on glycerol [26], which may explain the poor pool sizes of these three metabolites. The drops in levels of precursors for biosynthesis might be linked to decreased amino acid pools in the central metabolic pathway, leading to the reduced biomass production and *μ* in HT66LSP.

Shikimate pathway converts the primary carbon metabolites via shikimate to chorismate. DAHP, DHQ, DHS, and shikimate were more abundant in HT66LSP. Chorismate serves mainly as a common precursor for the syntheses of PCN, aromatic amino acids, folate, and co-enzyme Q. DHHA, the last stable intermediate in the pathway leading to PCA, was more abundant in HT66LSP. However, the level of PCA was lower. One of the possible explanations could be that PCA was more rapidly catabolized by PhzH which was transcriptionally upregulated than biosynthesized from DHPCA. The conversion of PCA to PCN was also shown to be essential for the biocontrol activity of strain *P. chlororaphis* PCL1391 [27]. The levels of other competing pathways metabolites derived from chorismate were lower in HT66LSP, including 4-aminobenzoate, folate, phenylalanine, tryptophan, and tyrosine. In addition, 4-hydroxybenzoate was below the detection level in both strains. These results indicated that manipulations of regulatory genes reduced competing drains on additional chorismate-utilizing pathways and promoted PCN biosynthesis.

NADPH provides the reducing power that drives numerous anabolic reactions, including those responsible for the biosynthesis of all major cell components in the central carbon metabolic pathway and the shikimate pathway. NADH is involved in ATP generation of oxidative phosphorylation. Compared with HT66, NADPH, NADH, NAD^+^, ATP, ADP, and AMP were higher, while NADP^+^ was lower in HT66LSP. The energy charges in HT66 and HT66LSP were 0.85 ± 0.05 and 0.84 ± 0.01, respectively, consistent with the previous reported values (0.80–0.95) [28].

### Qualitative assessment of dynamic labeling data with metabolomics data

**Fig. 5:**
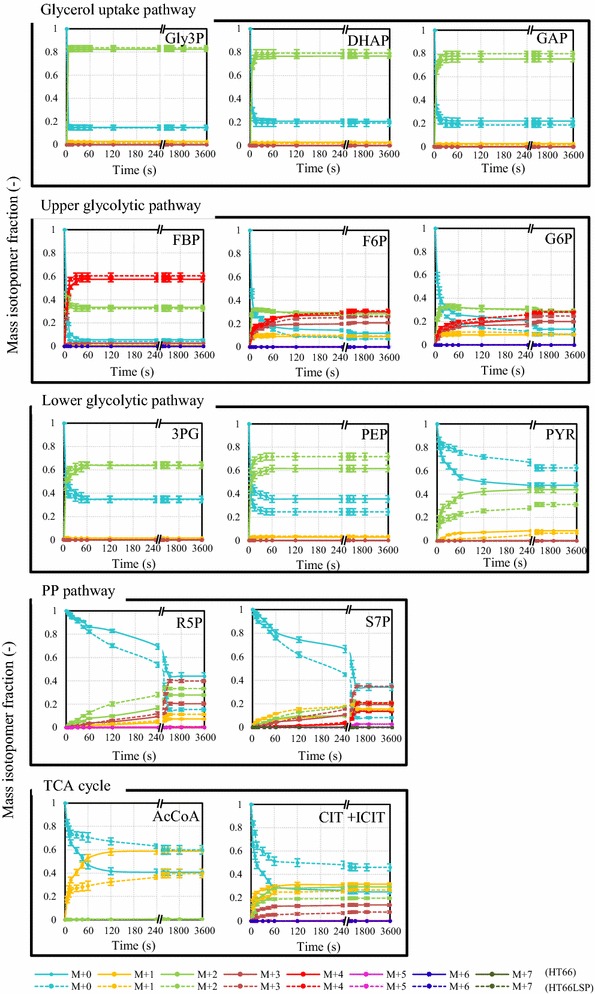
^13^C-labeling trajectories of selected intracellular metabolites after the introduction of [1,3-^13^C]glycerol at the exponential phase. Mass isotopomer data corrected for natural isotopic abundances are shown. The solid lines (HT66) and dashed lines (HT66LSP) illustrate the measured labeling trends

**Fig. 6:**
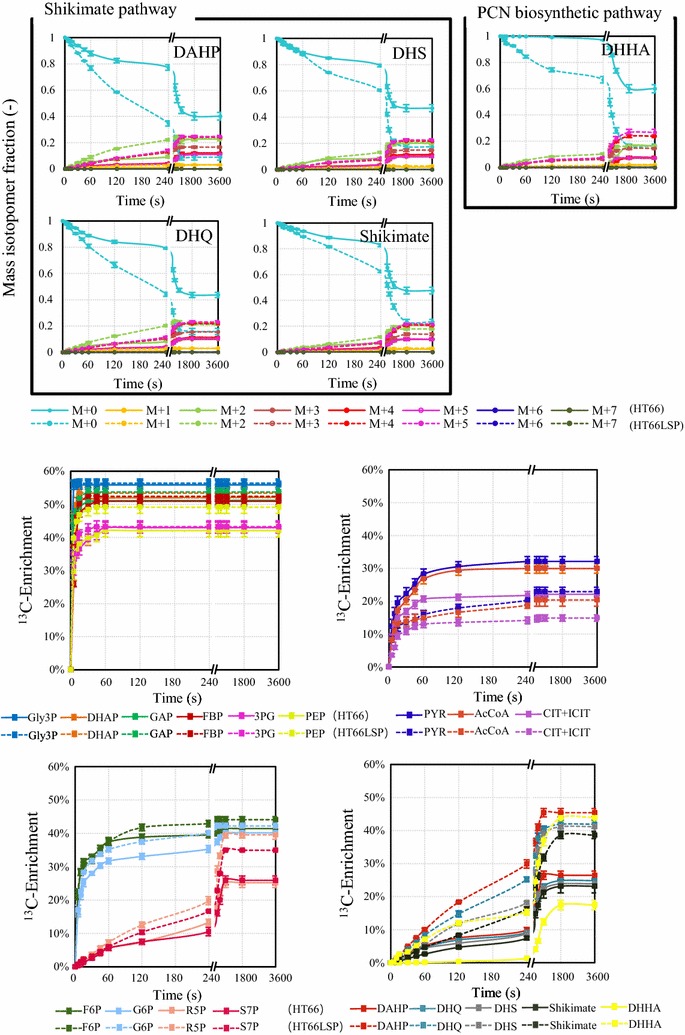
Average ^13^C-enrichments calculated using the formula $\left( {\frac{1}{N}} \right)\sum\nolimits_{i = 1}^{N} {Mi \times i}$, where *N* is the number of carbon atoms in the metabolite and *Mi* is the fractional abundance of the *i*th mass isotopomer. The solid lines (HT66) and dashed lines (HT66LSP) illustrate the measured labeling trends

To determine the relative pathway activities and help infer metabolic fluxes in response to the PCN overproduction, dynamic ^13^C-labeling experiments were performed, and integrated with metabolomics data to perform crossover analyses in HT66 and HT66LSP (Figs. 5, 6). Immediately after the introduction of [1,3-^13^C]glycerol or [U-^13^C]glycerol to the culture, the labeling dynamics of key metabolites were measured over a 1-h time window (Fig. 5; Additional file 2a). The average ^13^C-enrichment of each metabolite increased monotonically over time (Fig. 6; Additional file 2b), with the MDV shifting gradually toward heavier mass isotopomers. In addition to determining the level of enrichment, LC–MS analysis also provides information on the distribution of mass isotopomers of metabolites. The labeling dynamics were similar in two glycerol tracers.

The glycerol utilization pathway and glycolysis intermediates Gly3P, DHAP, GAP, 3PG, PEP, and FBP, distances of which from the entry point of the tracer is near, had the short response times, and a saturation in the labeling state was already reached within 60 s in both strains. The labeling dynamics of these metabolites were comparable with the exception of PEP in two strains. The identical labeling dynamics and intracellular concentrations of Gly3P, DHAP, and GAP indicated the similar flux through the glycerol utilization pathway in the two strains. In HT66LSP, the enrichment of PEP, which is the downstream metabolite of 3PG, was higher compared with 3PG throughout the labeling period, revealing unusual labeling patterns caused by metabolite channeling. A slight delay was found in the enrichment of metabolites in the other parts of central carbon metabolism, whereby isotopic stationary was reached within the time frame of 240 ≤ t ≤ 900 s. The two strains had the similar rates of labeling incorporation of F6P and G6P, together with intracellular concentrations, suggesting a lower flux through the upper glycolytic pathway or faster exchange with the nonoxidative PP pathway in HT66LSP. S7P and R5P which connect to the glycolytic pathway reached higher isotopic ratios more quickly in HT66LSP than in HT66. Slower decreases in M + 0 of PYR, AcCoA, and CIT + ICIT downstream of PEP were observed, together with their lower concentrations in HT66LSP than in HT66, signifying a lower flux through the lower glycolysis and the TCA cycle in the engineered strain.

Compared with glycolysis, the labeling dynamics in the shikimate pathway and PCN biosynthetic pathway were orders of magnitude slower, whereby isotopic stationary was reached within 30 min. A time lag of at least 30 s was observed before the labeling reached metabolites involved in these pathways followed by a further decrease in the labeling dynamics in both strains. HT66LSP had higher rates of labeling incorporation of DAHP, DHQ, DHS, shikimate and DHHA than HT66. Interestingly, shikimate was less labeled than its downstream product DHHA in HT66LSP, which implied the presence of metabolite channeling. In addition, distributions of labeled mass isotopomers of DAHP, DHQ, DHS, shikimate and DHHA were also different: predominantly either M + 2 ([1,3-^13^C]glycerol) or M + 3 ([U-^13^C]glycerol) in HT66 and either M + 5 ([1,3-^13^C]glycerol) or M + 7 ([U-^13^C]glycerol) in HT66LSP. The higher mass isotopomer of these metabolites formed in the condensation of a ^13^C-labeled PEP and a ^13^C-labeled E4P, while the lower mass isotopomer came from either ^13^C-labeled PEP or E4P. The faster labeling dynamics and heavier mass isotopomers integrating with higher levels of shikimate pathway intermediates in HT66LSP inferred a higher flux through this pathway.

## Discussion

### Glycerol utilization

In the glycerol uptake pathway, the gene transcript levels, pool sizes of metabolites, and the flux were not substantially affected by the PCN overproduction (Figs. 3, 4, 5 and 6; Additional file 2), which led to the constant glycerol consumption rate in HT66LSP (Table 1). This indicated the structural robustness of the metabolic network itself.

### Regulatory mechanisms involved in rerouting of central carbon to shikimate pathway

Shikimate pathway, a bridge between primary and secondary metabolism, links the carbohydrate metabolism to the biosynthesis of phenazines [29, 30]. The shikimate pathway starts with DAHP-synthase-mediated condensation of PEP and E4P into DAHP. This step is one of the primary limiting reactions for the whole pathway [31]. In the starting strain HT66, the labeling dynamics of DAHP were much slower than PEP (Figs. 5, 6; Additional file 2), suggesting a potential bottleneck at this step. DAHP synthase is a key enzyme controlling the shikimate pathway, which is supported by several genome-scale and enzymatic analyses of mutant strains with improved shikimate production, in which this enzyme has been found to be overexpressed [32, 33]. By modifying regulatory genes, we found that *phzC* encoding DAHP synthase was upregulated, accompanied with more abundances of intracellular metabolites and the increased flux through the shikimate pathway, finally translating into PCN overproduction in the engineered strain (Figs. 2d, 3, 4, 5, 6, and Additional file 2). Our research confirmed the biological function of DAHP synthase to regulate the flux from the central metabolic pathway toward the shikimate pathway.

PEP, the precursor of the shikimate pathway, is a key intermediate in the glycolytic pathway. The carbon flux of PEP to its derived compounds usually competes with that of the lower glycolysis and the TCA cycle [34]. Compared with HT66, the carbon partitioning at PEP node directed relatively less carbon into the lower glycolysis and the TCA cycle (Figs. 5, 6; Additional file 2), associated with the downregulation of the genes (*pykF*, *aceE*, *gltA*, *icd*, *sucA*, and *sucC*) involved in this pathway (Fig. 3) in HT66LSP. The results demonstrated that reducing the carbon flux below the PYR node had a clear benefit to the PCN production. Beckers et al. [35] reported that the reductive TCA cycle influenced the production of poly(3-hydroxyalkanoate) which was synthesized from PYR in *P. putida* KT2440 grown on glycerol adversely.

Metabolomics analysis revealed that a handful of metabolites in the central carbon metabolism were less abundant, together with increases in the shikimate pathway intermediates in HT66LSP (Fig. 4), indicating significant drains on central carbon to provide precursors for the shikimate pathway and again confirming effective manipulations.

PCN production starts from chorismate [29], and the shikimate pathway converts central carbon metabolites via shikimate to chorismate [31], further away from glycerol consumption. Thus, metabolic engineering of the shikimate pathway and the PCN biosynthetic pathway alone maybe insufficient for increasing the yield and productivity from glycerol. Thus, genetic modifications to alter central carbon metabolism to supply the needed precursors are required. To improve E4P availability, the overexpressions of *tktA* and *talA* in the PP pathway are a successful strategy for the optimization of shikimate-producing strains [36], which can be applied for the efficient production of PCN. For the PEP supply, since metabolic flux through the TCA cycle is theoretically not needed for shikimate production [37], inactivation of pyruvate kinase represents a potential target to further enhance PCN production, which has been successively applied in its upstream metabolites chorsimate and PCA [15, 38].

### Cofactors and energy metabolism

Compared with HT66, the NADPH, NADH, and ATP were all present in lower concentrations in HT66LSP (Fig. 4), which can be explained by the following reasons: (1) the lower flux from PYR toward the TCA cycle yielded less cofactors and ATP; (2) the conversion from DHS to shikimate consumed more NADPH; (3) S3P formation from shikimate needed more ATP; (4) the expression levels of *zwf* and *gnd* were not substantially modified, which may not generate more NADPH. The results indicated that the PCN production is constrained to a greater extent by precursor availability than by cofactors and ATP-deficiency.

### Effective synthesis of PCN through metabolite channeling

Metabolite channeling, also known as substrate channeling, is a process of direct transfer of the product of an enzyme to another proximate enzyme or cell as its substrate without equilibration with the bulk phase [39]. Zhao et al. [40] showed that the chemotactic assembly of enzymes occurs even under cytosolic crowding conditions, which indicates that all native enzymes can naturally form a channel. Isotope dilution and enrichment has proved useful in measuring metabolite channeling [41–43]. In this study, we noted the metabolite channeling of 3PG into PEP and shikimate into DHHA in HT66LSP by this method (Figs. 5, 6; Additional file 2). In previous studies, metabolite channeling has been reported in the glycolytic pathway [43, 44], the Calvin cycle [45], the oxidative PP pathway [46], the Calvin–Benson–Bassham cycle [41], and so on. Our results provide the first evidence of metabolic channeling in the PCN biosynthetic pathway.

The effect of channeling could significantly improve the reaction thermodynamics and accelerated reaction rates by enzyme complexes [47]. In this research, metabolite channeling could enable high-yielding synthesis of PCN. First, PEP and DHHA would be synthesized more efficiently with high catalytic rates. Moreover, lower levels of metabolites in additional chorismate-utilizing pathways in HT66LSP indicated the forestallment of the chorismate competition among different pathways through channeling of shikimate into DHHA. In addition, when shikimate was directed toward DHHA, the unstable chorismate might be protected. By mimicking natural enzyme complexes for metabolite channeling, the strategy of structuring nonnatural cascade reactions has also made considerable success in the fields of metabolic engineering and synthetic biology, such as co-localization of cascade enzymes by protein, nucleic acid, and polymer scaffolds [48–50]. The downstream metabolites of DHHA in the phenazine biosynthetic pathway are not stable [51]. In addition, AOCHC is likely toxic to the cell so that its accumulation must be prevented [51]. To overcome these obstacles, co-localization of the core phenazine biosynthetic enzymes (PhzA, PhzB, PhzF, and PhzG) via protein scaffold would increase the robustness of the PCN biosynthetic pathway and prevent this pathway from releasing intermediates. The channeling strategy would have great biotechnological potentials for the overproduction of PCN.

## Conclusions

In this work, we obtained a PCN-overproducing strain through knocking out three regulatory genes, *lon*, *pars*, and *psrA* in *P*. *chlororaphis* HT66. The engineered strain produced 4.10 g/L of PCN with a yield of 0.23 (g/g) from glycerol, which was the highest PCN titer and the yield ever obtained so far among PCN-producing strains. The integrated analysis of gene expression, metabolomics, and dynamic ^13^C tracer enabled us to gain a more in-depth insight into complex mechanisms for the PCN overproduction. Rerouting of central carbon to the shikimate pathway and metabolite channeling facilitated the PCN production. This study demonstrated that integrating systems biology analysis could be an efficient way to identify metabolic responses to the PCN overproduction, forming the basis for the rational design and engineering of the high efficiency strains using glycerol as the cost-effective carbon source. This approach would have a great potential for the future industrial production and agricultural application of biopesticide phenazines.

## Methods

### Strains, culture medium, and growth conditions

The strains, plasmids, and primers used in this study are listed in Table 2. The wild-type strain *P. chlororaphis* HT66 (CCTCC, M2013467) was obtained from China Center for Type Culture Collection. For genetic manipulations, *E. coli* strains were grown at 37 °C in LB medium. *P*. *chlororaphis* HT66 and its derivative strains stored in a − 80 °C freezer were activated at 28 °C for 12-24 h in King’s B agar media (glycerol 18 g/L, tryptone 20 g/L, MgSO_4_ 0.732 g/L, K_2_HPO_4_ 0.514 g/L). Selection of single colonies from Petri plates was performed, which were then used to inoculate 10 mL of King’s B broth in 50 mL flasks. Cultures were then grown at 28 °C in a flask kept under stirring at a speed of 180 rpm overnight. Portions of these cultures were then inoculated into 500-mL baffled flasks containing 120 mL King’s B broth to achieve an initial OD_600_ = 0.02. If necessary, ampicillin (100 μg/mL) and kanamycin (50 μg/mL) were added into the culture medium of *E. coli* and *P*. *chlororaphis*. After 8–36-h growth at 28 °C and 180 rpm, cultures were collected for the measurements of cell growth, glycerol, and PCN.

**Table 2:** Strains and plasmids used in this study

Strains and plasmids	Relevant genotype or description	Source or reference
Strains *E. coli* DH5α	*E. coli* F^−^Ф80*lacZ*ΔM15 Δ(*lacZYA*-*argF*) M15 U169 *recA1 endA1 hsdR17* (rk^−^ mk^−^) *phoA supE44 thi*^−1^ *gyrA96* relA1	Invitrogen
*E. coli* S17-1 (λpir)	res^−^ pro mod^+^ integrated copy of RP4, mob^+^, used for incorporating constructs into *P*. *chlororaphis*	[58]
*P*. *chlororaphis* HT66	*P. chlororaphis* HT66 Wild-type, Ap^r^ Sp^r^	China Center for type culture collection
HT66L	*lon* in-frame deletion mutant of HT66	This study
HT66LS	*parS* in-frame deletion mutant of HT66L	This study
HT66LR	*parR* in-frame deletion mutant of HT66L	This study
HT66LSP	*psrA* in-frame deletion mutant of HT66LS	This study
Plasmids pK18mobsacB	Broad-host-range gene replacement vector; *sacB*, Kan^r^	[59]
pK18-lon	pK18mobsacB containing *lon* flanking region	This study
pK18-parS	pK18mobsacB containing *parS* flanking region	This study
pK18-parR	pK18mobsacB containing *parR* flanking region	This study
pK18-psrA	pK18mobsacB containing *psrA* flanking region	This study

### Construction of nonscar deletion mutant strains

The *lon* gene involved in the biosynthesis of PCN in *P. chlororaphis* HT66 was disrupted using the nonscar deletion method as described by Du et al. [52]. Briefly, two DNA fragments flanking the upstream and downstream area were amplified by PCR from the genome and combined using overlap PCR. The nonscar-modified DNA fragment was cloned into pK18mobsacB, and the resulting plasmid was then transferred into *E. coli* S17-1 (λpir) to be mobilized into HT66 by biparental mating. The colones occurring as a single-crossover event were selected from plates containing 100 μg/mL of ampicillin and 50 μg/mL of kanamycin, and then clones occurring as a double-crossover events were selected from plates containing 15% (w/v) sucrose and 50 μg/mL of kanamycin [6]. The mutant strain HT66L was further verified by PCR and DNA sequencing. In similar ways, the *parS, parR*, and *prsA* nonscar-deleted mutants were constructed in their corresponding strains. All the primers used to delete genes are shown in Additional file 3.

### Analytical methods

Dry cell weight (DCW) in King’s B medium was calculated from the optical density at 600 nm (1 OD_600_ = 0.4114 g DCW L^−1^). Concentrations of glycerol were measured by high-performance liquid chromatography (HPLC) (model 1260, Agilent, Santa Clara, USA) using a cation-exchange column (HPX-87H, Bio-Rad, Hercules, CA) and a differential refractive index (RI) detector. A mobile phase of 5 mM H_2_SO_4_ at 0.5 mL/min flow rate was used, and the column was operated at 60 °C. In order to extract PCN, fermentation broth (400 μL) was acidified to pH 2.0 with 6 M HCl, and then 3.6 mL of ethyl acetate was added. The sample was vigorously agitated and centrifuged at 13,000×*g* for 5 min. A 400-μL portion of the organic layer was collected and evaporated. The residue was dissolved in 1 mL of acetonitrile. Concentrations of PCN were measured by HPLC using a C18 reverse-phase column (Agilent Eclipse XDB-C18, 4.6 mm × 250 mm, 5μΜ, Santa Clara, USA) by means of a UV light detector [6]. A mobile phase of 8% acetonitrile and 92% 5 mM NH_4_Ac at 1 mL/min flow rate was used, and the column was operated at 30 °C.

### Quantitative real-time PCR (qRT-PCR) analysis

Culture samples at the mid-logarithmic growth phase were collected for RNA extraction. Total RNA was isolated using an RNA Extraction Kit (ABigen Corporation, China). Contaminating DNA was removed with RNase-free DNase I (ABigen Corporation, China). The first-strand cDNA was synthesized using PrimeScript™ II 1st Strand cDNA Synthesis Kit (Takara Co. Ltd., China). QRT-PCR was performed with the SYBR^®^ Premix Ex Taq™ Kit (Takara Co. Ltd., China) on an ABI Stepone Real-Time PCR System (Applied Biosystems, USA). The primers used for qRT-PCR are listed in Additional file 1. The PCR conditions were 95 °C for 4 min, followed by 35 cycles of denaturation at 95 °C for 15 s, annealing at 57 °C for 15 s, and extension at 72 °C for 20 s. Three biological samples were analyzed, and each sample was analyzed three times. The housekeeping gene 16S rRNA was used as an internal standard. Fold changes of genes of interest were calculated as 2^−ΔΔCT^ according to Schmittgen and Livak [53]. The data were averaged and presented as the mean ± standard deviation. Significant differences were determined by one-way analysis of variance (ANOVA). Statistical significance was defined as *p* < 0.05.

### Sampling, quenching, and extraction of intracellular metabolites

Samples for intracellular metabolome analysis were withdrawn from the mid-logarithmic growth phase. For quenching and extraction, the protocol was adapted from the works of Millard et al. [54] and Toya et al. [55] with slight modifications. In brief, 10 mL of culture was poured into 50-mL falcon-tube containing 5 mL of 0.9% NaCl solution precooled at 0 °C, and the tube was immediately immersed in liquid N_2_. The sample solutions were manually agitated using a digital thermometer, and then it could be cooled to 0 °C within 10 s. Extracellular medium was removed by centrifugation at 8000 rpm at 0 °C for 3 min. To accurately determine the metabolites’ concentrations, isotope dilution mass spectrometry (IDMS) [56] was adopted in this study. After quenching, the cell pellet was suspended in 5 mL of cooled methanol containing U-^13^C-labeled cell extract as internal standards. After 30 s of sonication, 4 mL of chloroform and 1.6 mL of Milli-Q water were added to the solution and mixed, and the mixture was centrifuged at 4600×*g* at 4 °C for 5 min. The methanol layer was filtered by centrifugation through a 5-kDa cutoff filter (Merck Millipore Ltd., Darmstadt, Germany) to extract most of the intracellular metabolites, with the exception of 4-aminobenzoate, 4-hydroxybenzoate, folate, and PCA, which were extracted from the chloroform layer. The filtrate was thoroughly lyophilized (FreeZone 6 Liter, Labconco, USA) and reconstituted in 50 μL of methanol–water (1:1, v/v) just prior to measurement.

## ^13^C-pulse experiments

A sample of corresponding to time zero (unlabeled) was withdrawn just before the ^13^C-pulse experiments. After 23.6 h, labeling experiments were started by the rapid addition of 165.1 mM of either 100% [1,3-^13^C]glycerol (99% purity; Cambridge Isotope Laboratories Inc., Andover, MA) or 100% [U-^13^C]glycerol (99% purity; Cambridge Isotope Laboratories Inc., Andover, MA) to 30.4 mM unlabeled glycerol present in the flask, leading to a final glycerol concentration of 195.5 mM. To measure the ^13^C-incorporation into intracellular metabolites, 10 mL samples were withdrawn and rapidly quenched at time points of 5, 10, 15, 30, 45, 60, 120, 240, 450, 600, 900, 1800, and 3600 s, each in three biological replicates.

### LC–MS measurement of metabolite labeling and concentration

Metabolite samples were analyzed via high resolution mass spectrometry analysis, carried on a Waters I-Class Acquity UPLC (Waters, UK) coupled with a Vion IMS QToF (Waters, UK) using a SeQuant ZIC-HILIC column (100 mm × 2.1 mm i.d., 3.5 µm) (Merck, Germany). The mobile phase A was 50 mM ammonium formate in water, and mobile phase B was acetonitrile. Metabolites were separated via gradient elution under the following conditions: 0–10 min, 90–50% B; 10–12 min, 50–90% B; 12–15 min, 90% B; and the column was maintained at 45 °C. The flow rate was 0.4 mL/min. The parameters of high-resolution mass spectrometry analysis on full scan mass spectrometry were as follows: MS range, *m/z* 50–1000; scan time, 0.3 s; CE 6 eV; desolvation temperature, 500 °C; source temperature, 120 °C; desolvation gas, 1000 L/h; cone gas, 50 L/h; capillary voltage, 2000 V. The lock correction (lock sprayer reference: mass, 556.2766 *m/z*; interval, 0.5 min; sample time, 0.5 min; CE, 6 eV; flow rate 10 μL/min) enabled isotopic *m/z* screen, with tolerance of 3 mDa mass error, the expected adduct –H. Data were acquired and processed using UNIFI 1.8.1. All purified metabolite standards were purchased from Sigma-Aldrich (St. Louis, MO, USA), with the exception of DHHA, which was produced in our own lab [57]. Quantitation of the peaks was achieved by comparing peak areas with the standards.

Significant differences were determined by a two-tailed Student’s *t* test using Microsoft Excel 2013 program. Statistical significance was defined as *p* < 0.05. Metabolite mass isotopomer distribution was determined based on the ratio of the integrated peak areas of the chosen isotopomer to the sum of all the integrated peak areas of the possible isotopomers for the given metabolites.

## Abbreviations

### Metabolites

AcCoA: acetyl-CoA; 4ABA: 4-aminobenzoate; ADIC: 2-amino-4-deoxychorismic acid; AKG: α-ketoglutarate; AOCHC: 6-amino-5-oxocyclohex-2-ene-1-carboxylic acid; 1,3 BPG: 1,3-bisphosphoglyceric acid; CHOR: chorismate; CIT: citrate; DAHP: 3-deoxy-D-arabino-heptulosonate 7-phosphate; DHAP: dihydroxyacetone phosphate; DHHA: trans-2,3-dihydro-3-hydroxyanthranilic acid; DHPCA: 5,10-dihydrophenazine-1-carboxylic acid; DHQ: 3-dehydroquinate; DHS: 3-dehydro-shikimate; E4P: erythrose 4-phosphate; F6P: fructose 6-phosphate; FBP: fructose 1,6-bisphosphate; FUM: fumarate; G6P: glucose-6-phosphate; GAP: glyceraldehyde 3-phosphate; GLX: glyoxylate; Gly3P: glycerol-3-phosphate; HHPDC: hexahydrophenazine-1,6-dicarboxylic acid; ICIT: isocitrate; KDPG: 2-keto-3-deoxy-6-phosphogluconate; MAL: malate; OAA: oxaloacetate; 2PG: 2-phosphoglyceric acid; 3PG: 3-phosphoglyceric acid; 6PG: 6-phosphogluconate; 6PGNL: 6-phosphogluconolactone; PCA: phenazine-1-carboxylic acid; PEP: phosphoenolpyruvate; Phe, phenylalanine; PYR: pyruvate; R5P: ribose 5-phosphate; RU5P: ribulose 5-phosphate; S3P: shikimate 3-phosphate; S7P: sedoheptulose 7-phosphate; SUCCoA: succinyl-CoA; SUC: succinate; THPCA: tetrahydrophenazine-1-carboxylic acid; Trp, tryptophan; Tyr, tyrosine; X5P: xylulose 5-phosphate.

### Proteins (enzymes)

PhzA: phenazine biosynthesis protein PhzA; PhzB: phenazine biosynthesis protein PhzB; PhzF: phenazine biosynthesis protein PhzF family; PhzG: pyridoxamine 5′-phosphate oxidase.

## Additional files

**Additional file 1.** Culture profiles of HT66LR.

**Additional file 2.** ^13^C-labeling trajectories of selected intracellular metabolites after the introduction of [U-^13^C]glycerol at the exponential phase. **a** Mass isotopomer abundances after correction for natural isotopic abundances. **b** Average ^13^C-enrichments calculated using the formula $\left( {\frac{1}{N}} \right)\sum\nolimits_{i = 1}^{N} {Mi \times i}$, where *N* is the number of carbon atoms in the metabolite and *Mi* is the fractional abundance of the *i*th mass isotopomer. The solid lines (HT66) and dashed lines (HT66LSP) illustrate the measured labeling trends.

**Additional file 3.** Primers used in this study.
