# Supplementary figures and images for: Engineering and systems-level analysis of Pseudomonas chlororaphis for production of phenazine-1-carboxamide using glycerol as the cost-effective carbon source
Source: Biotechnol Biofuels. 2018 May 4;11:130. doi: 10.1186/s13068-018-1123-y (PMC5934903; doi:10.1186/s13068-018-1123-y)

**Additional file 1. Culture profiles of HT66LR.**

*P.chlororaphis* HT66LR

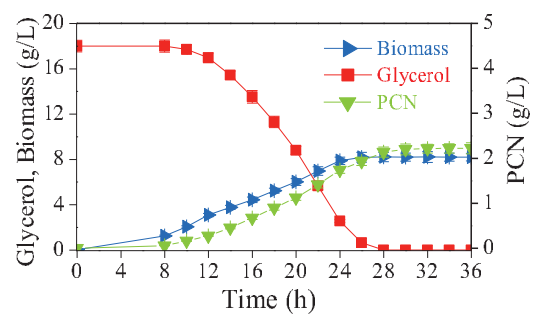

Supplement: Supplementary file 1 — Additional file 1. Culture profiles of HT66LR. [file 13068_2018_1123_MOESM1_ESM.pdf]
