# Supplementary material for: Engineering and systems-level analysis of Pseudomonas chlororaphis for production of phenazine-1-carboxamide using glycerol as the cost-effective carbon source
Source: Biotechnol Biofuels. 2018 May 4;11:130. doi: 10.1186/s13068-018-1123-y (PMC5934903; doi:10.1186/s13068-018-1123-y)
Supplement: Supplementary file 2 — Additional file 2. 13C-labeling trajectories of selected intracellular metabolites after the introduction of [U-13C]glycerol at the exponential phase. a Mass isotopomer abundances after correction for natural isotopic abundances. b Average 13C-enrichments calculated using the formula $\left( {\frac{1}{N}} \right)\sum\nolimits_{i = 1}^{N} {Mi \times i}$1N∑i=1NMi×i, where N is the number of carbon atoms in the metabolite and Mi is the fractional abundance of the ith mass isotopomer. The solid lines (HT66) and dashed lines (HT66LSP) illustrate the measured labeling trends. [file 13068_2018_1123_MOESM2_ESM.pdf]

Additional file 2. ^{13}C-labeling trajectories of selected intracellular metabolites after the introduction of [U-^{13}C]glycerol at the exponential phase. **a** Mass isotopomer abundances after correction for natural isotopic abundances. **b** Average ^{13}C-enrichments calculated using the formula $(\frac{1}{N})\sum_{i=1}^{N} Mi \times i$, where N is the number of carbon atoms in the metabolite and Mi is the fractional abundance of the ith mass isotopomer. The solid lines (HT66) and dashed lines (HT66LSP) illustrate the measured labeling trends.

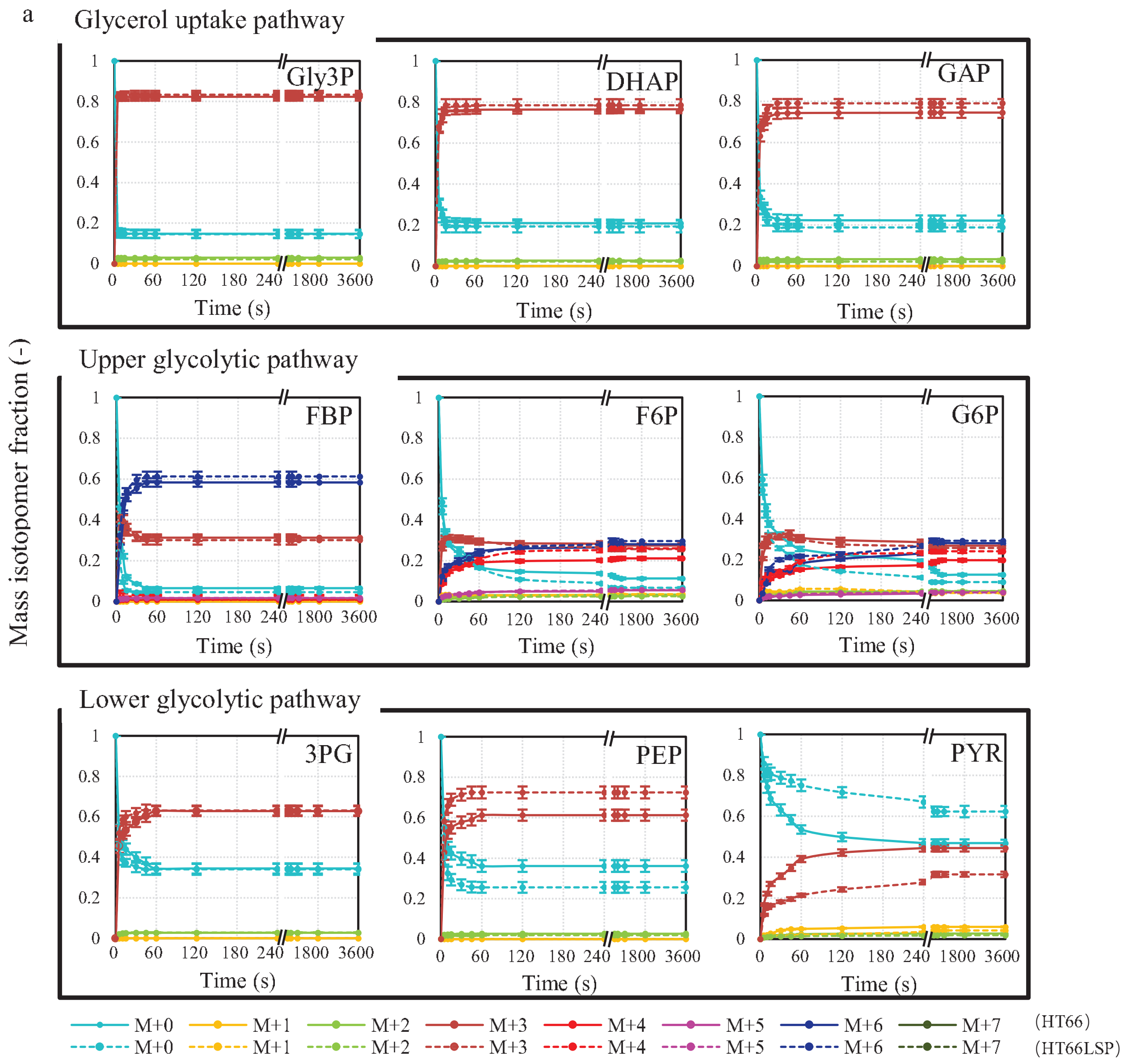

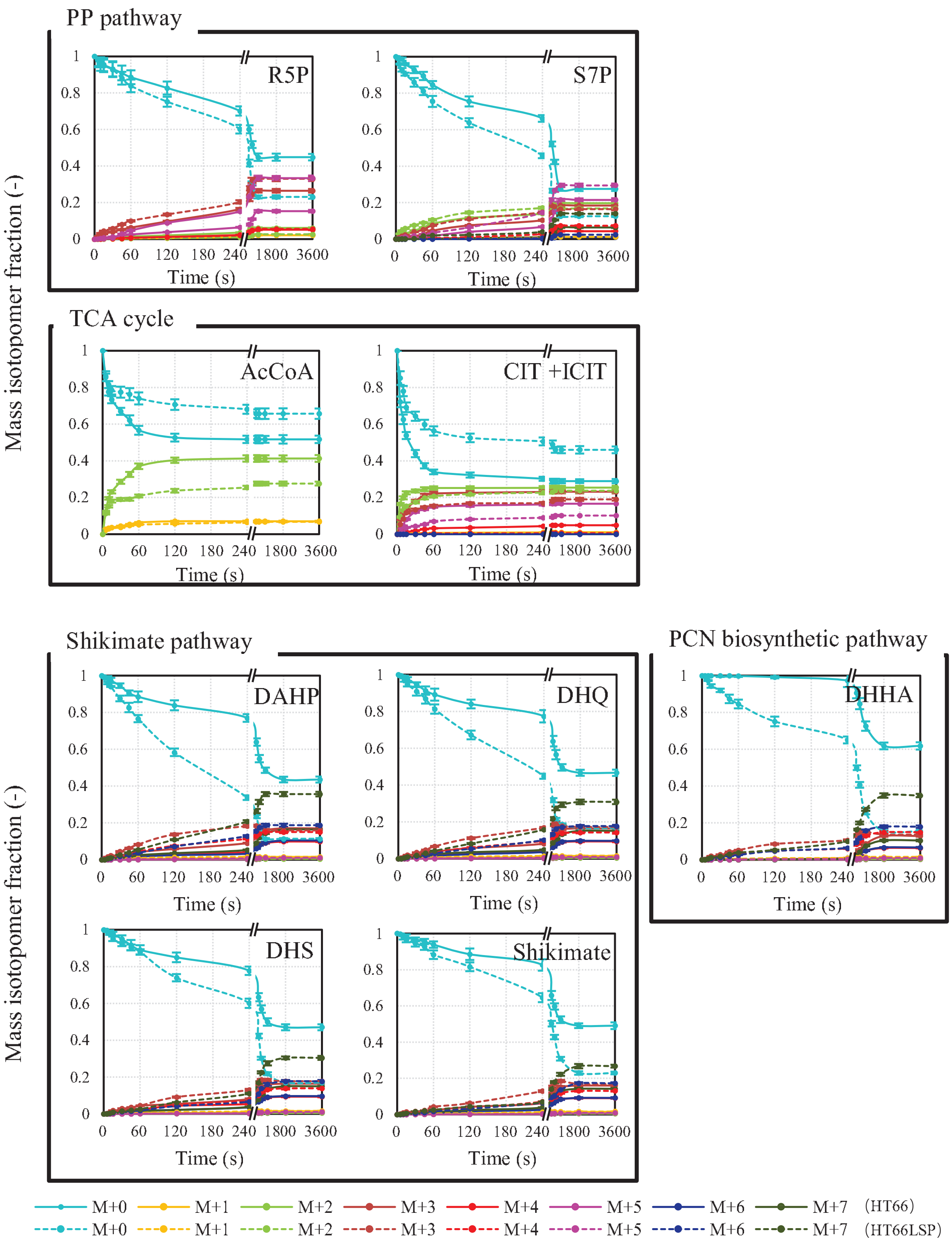
PP pathway
R5P
S7P
TCA cycle
AcCoA
CIT +ICIT
Shikimate pathway
DAHP
DHQ
DHS
Shikimate
PCN biosynthetic pathway
DHHA
Mass isotopomer fraction (-)
Time (s)
0 60 120 180 240 1800 3600
0 0.2 0.4 0.6 0.8 1
M+0 M+1 M+2 M+3 M+4 M+5 M+6 M+7 (HT66)
M+0 M+1 M+2 M+3 M+4 M+5 M+6 M+7 (HT66LSP)

b

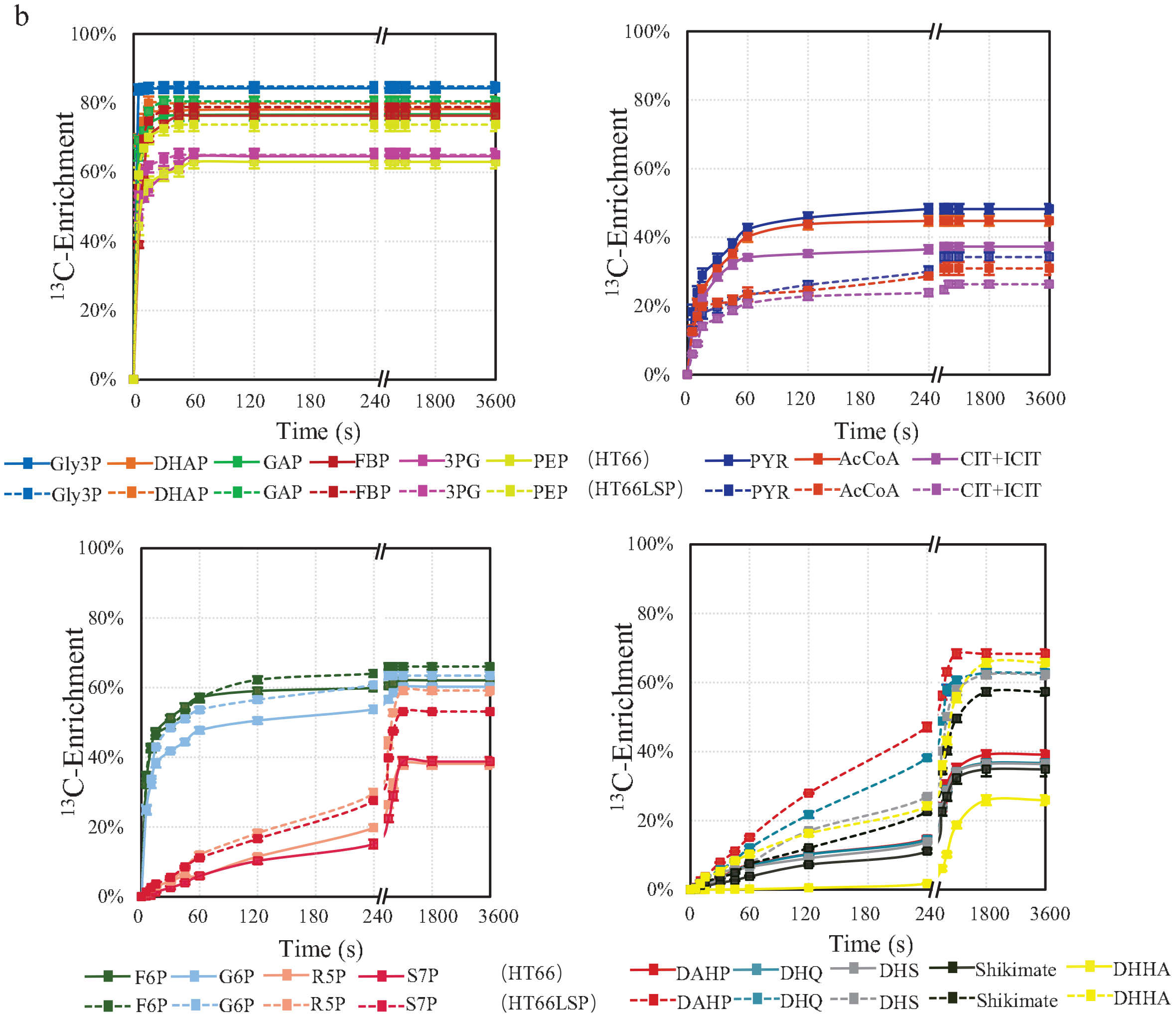
^{13}C-Enrichment
Time (s)
0% 20% 40% 60% 80% 100%
0 60 120 180 240 1800 3600
Gly3P DHAP GAP FBP 3PG PEP (HT66)
Gly3P DHAP GAP FBP 3PG PEP (HT66LSP)
PYR AcCoA CIT+ICIT
PYR AcCoA CIT+ICIT
F6P G6P R5P S7P (HT66)
F6P G6P R5P S7P (HT66LSP)
DAHP DHQ DHS Shikimate DHHA
DAHP DHQ DHS Shikimate DHHA
