# Supplementary material for: Engineering and systems-level analysis of Pseudomonas chlororaphis for production of phenazine-1-carboxamide using glycerol as the cost-effective carbon source
Source: Biotechnol Biofuels. 2018 May 4;11:130. doi: 10.1186/s13068-018-1123-y (PMC5934903; doi:10.1186/s13068-018-1123-y)
Supplement: Supplementary file 3 — Additional file 3. Primers used in this study. [file 13068_2018_1123_MOESM3_ESM.pdf]

**Additional file 3** Primers used in this study.

| Primer | Sequence |
|---|---|
| Primers for gene deletion | |
| *lon*-F1 | CGGAATTCCGACTGCTGGTTTGACAGAAGG |
| *lon*-R1 | GGATAACCATGTGCGGATAA |
| *lon*-F2 | TTATCCGCACATGGTTATCCTCCTTGACAGCTTTTTAGAGC |
| *lon*-R2 | GCTCTAGAGCCCAGAAACGGTTGTACTTCAA |
| *parS*-F1 | CGGGATCCCGATGGATAACCCGGGTCTTGG |
| *parS*-R1 | CTAGAGCTCCCACGCGAATG |
| *parS*-F2 | CATTCGCGTGGGAGCTCTAGAGGTCGAGGTGACGGATTCG |
| *parS*-R2 | CCCAAGCTTGACTTCTGACGCTATCGCGGAAC |
| *parR*-F1 | GAGAGGATCCCACCAGCGCAGCGAGAAAGACATAG |
| *parR*-R1 | AACAGATAGCCCTTGCCCCAAAACCGTGTTGGGACAGGAA |
| *parR*-F2 | TGGGGCAAGGGCTATCTGTT |
| *parR*-R2 | GAGAAAGCTTCTGGACGAAGAACTGGGCAA |
| *psrA*-F1 | GCGGGATCCGGTCCAAACCGACTGCCAACA |
| *psrA*-R1 | CAGGTGCGTGAGCTGAGTGC |
| *psrA*-F2 | ACTCAGCTCACGCACCTGATGGCTACTCCGCCTGACAAA |
| *psrA*-R2 | CCCAAGCTTCGGATTGATGTTGCAGGACTCTTC |
| Primers for qRT-PCR | |
| *glpK*-F | TGCACCATGACCAGGTAGCC |
| *glpK*-R | GCCAGACGATGGCGTTGTAG |
| *glpD*-F | CGACCTGAAACTGGACTCGC |
| *glpD*-R | ACGATGCGCTGGTCTTCATT |
| *tpiA*-F | AGCCATTCGTGAGCAGTTGG |
| *tpiA*-R | GAACAGTTCGACCGCATTGG |
| *zwf*-F | CTGGAGGAGGAACATGCGAA |
| *zwf*-R | TGCCGGTACGCAGGTAGAAC |
| *gnd*-F | TCACAACGGCATCGAGTACG |
| *gnd*-R | GACGTTGAGATCGAAGCGCT |
| *pykF*-F | GGACTACGTGGCCGTTTCCT |
| *pykF*-R | CGCTCGATCTTGGCTACCAG |
| *aceE*-F | TGAAGACCGCGCTCATTACC |
| *aceE*-R | CGGAATGGTGTTGCGATAGG |
| *gltA*-F | TTTTAACCGGCACCGTTGGT |
| *gltA*-R | GCAGGAGGCAGTCGACATGA |
| *icd*-F | GCGTACCGAAGAACCCGATC |
| *icd*-R | ATGCTTTTTCCACTGCAGCG |
| *sucA*-F | TGCTGCCACACGGTTATGAA |
| *sucA*-R | GGCATGCAGACCTGGATGTT |
| *sucC*-F | ACTGGTCAACATCTTCGGCG |
| *sucC*-R | GCCTTCAAGGCGAACAACAA |
| *gacA*-F | TCAAGTCATTCCAGCCGTCC |
| *gacA*-R | TCTGGCAGCCGACAATCATC |
| *gacS*-F | GGGCGGTTATTTCACCTGGA |
| *gacS*-R | GATACGTTCCAGTAGGGCGG |
| *phzI*-F | CAGCACCATGGAGCGCTATT |
| *phzI*-R | CGAGAGTTTGATGGCGAGGA |

| | |
|---|---|
| *phzR*-F | CGGTGAAGCACAGCAAAGTC |
| *phzR*-R | GCAAACGCCTTGGTCATCAG |
| *phzA*-F | GTACATGCGCACTAACGGCA |
| *phzA*-R | AGCATTTCTCCAGCCATTCG |
| *phzB*-F | GCGCCGCCATGAACTATTTA |
| *phzB*-R | AAACAGCATGTTCGGCCAAC |
| *phzC*-F | TTGGCGATGTTGAATTGCCG |
| *phzC*-R | CAGGCCGGTGAACCTATGAG |
| *phzD*-F | CGACCTGCCCATCAACCTC |
| *phzD*-R | CCACTTGTTCACGCAGGG |
| *phzE*-F | CCGACGAGCTGTACATGGTG |
| *phzE*-R | GCGCCATTTCCTTGAGGTAA |
| *phzF*-F | ACCGTGCCTTGTGCAACTTC |
| *phzF*-R | ATCCTCGACCACCCCATAGG |
| *phzG*-F | TGAAGAGCTCACGGATGTCG |
| *phzG*-R | TCAAGGCGCAACTCAAACAC |
| *phzH*-F | GGCGAGGTGTACAACCACGA |
| *phzH*-R | CAGATAGGCGTGCAGGACCA |
